# Novel Models of Crohn’s Disease Pathogenesis Associated with the Occurrence of Mitochondrial Dysfunction in Intestinal Cells

**DOI:** 10.3390/ijms23095141

**Published:** 2022-05-05

**Authors:** Alexander Blagov, Elena B. Zhigmitova, Margarita A. Sazonova, Liudmila M. Mikhaleva, Vladislav Kalmykov, Nikolay K. Shakhpazyan, Varvara A. Orekhova, Alexander N. Orekhov

**Affiliations:** 1 Laboratory of Angiopathology, Institute of General Pathology and Pathophysiology, 125315 Moscow, Russia; al.blagov2014@yandex.ru (A.B.); daisy29@mail.ru (M.A.S.); xxor2011@gmail.com (V.K.); 2 Federal State Budgetary Scientific Institution “Petrovsky National Research Centre of Surgery, A.P. Avtsyn Research Institute of Human Morphology”, 117418 Moscow, Russia; lenae7777@gmail.com (E.B.Z.); mikhalevalm@yandex.ru (L.M.M.); nshakhpazyan@gmail.com (N.K.S.); 3 Skolkovo Innovative Center, Institute for Atherosclerosis Research, 121609 Moscow, Russia

**Keywords:** Crohn’s disease, inflammation, mitochondrion

## Abstract

Crohn’s disease remains one of the challenging problems of modern medicine, and the development of new and effective and safer treatments against it is a dynamic field of research. To make such developments possible, it is important to understand the pathologic processes underlying the onset and progression of Crohn’s disease at the molecular and cellular levels. During the recent years, the involvement of mitochondrial dysfunction and associated chronic inflammation in these processes became evident. In this review, we discuss the published works on pathogenetic models of Crohn’s disease. These models make studying the role of mitochondrial dysfunction in the disease pathogenesis possible and advances the development of novel therapies.

## 1. Introduction

Crohn’s disease (CD) is a chronic disease that belongs to the group of inflammatory bowel diseases (IBD). Along with ulcerative colitis (UC), CD occupies one of the leading positions among other diseases of the digestive system in terms of the disease severity, the frequency of complications and the number of fatal cases [1]. CD can affect any part of the digestive system from the oropharynx to the anus and can affect all layers of the intestine [1]. The inflammatory process in the affected intestine is not homogeneous, with healthy areas of tissue alternating with affected sites [1]. Complications arising in CD can affect not only the gastrointestinal tract, but also other organs: the eyes, joints, skin, and liver [2]. Current therapy for CD is mainly aimed at reducing excessive inflammatory response.The main types of drugs used for treatment of this disease are 5-aminosalicylic acid derivatives, cytostatics, corticosteroid hormones, and monoclonal antibodies [3,4,5].

Crohn’s disease is most common in economically developed countries, with the United States and the United Kingdom being the leaders with the highest CD prevalence rates [1]. That makes CD one of the major challenges of public health in countries with high GDP. An additional feature of CD prevalence is the increased incidence of this disease among people aged 20–40 years, the most active and able-bodied period of life, which leads to significant economic impact of this condition. Moreover, the disease can have a heavy psychologic impact on the patient [4]. Available data show that CD is more common among the residents of large cities than in people living in the countryside [1], which indicates the possibility of an environmental factor in the CD development. It has been proven that mutations of certain genes may be responsible for the occurrence of CD [5], which, however, is not the only etiological factor of the disease.

Currently, the dominant hypothesis of CD pathogenesis is the development of an aggressive immune response to the gut microbiome in genetically predisposed individuals [6]. The resulting acute inflammation affects the intestinal wall and moves into a chronic phase over time [6]. Identifying different pathways for CD development is an important step in the fight against the disease. One likely cause of chronic inflammation is mitochondrial dysfunction. The possible role of mitochondrial dysfunction has been shown in the chronification of inflammation for diseases such as type 1 diabetes mellitus [7] and atherosclerosis [8]. Mitochondrial dysfunction was also described in CD [9]. In this review, we will focus on the possible role of mitochondrial dysfunction as one of the factors in CD pathogenesis and propose the models of CD pathogenesis.

## 2. Etiology of Crohn’s Disease

Among the causes of CD, the exceptional factor responsible for the development of the disease cannot be distinguished. Most likely, the etiology of CD is associated with the interaction of several factors, which complicates the understanding of the initial stages of the given disease pathogenesis. However, to date, several key etiological factors of CD development have been identified: nutrition, smoking, taking some types of drugs, gene mutations and intestinal dysbiosis.

### 2.1. Diet

The increased prevalence of CD in economically developed countries may be linked to lifestyle, particularly the diet primarily followed in these countries [10]. Epidemiological studies have shown that the American diet with a high intake of fats and carbohydrates and a low intake of fiber led to the increased risk of CD development [11]. In addition, dietary antigens along with bacterial antigens are the most common in the intestine, which supports the evidence of the diet’s contribution to CD development [11]. Changes in gene expression, modification of gut microbiome composition and effect on the intestine wall permeability can be identified among possible mechanisms of CD development exerted by food antigens [12]. Additionally, an unhealthy diet can lead to obesity, which increases the risk of CD [11]. In one study [13], CD patients were found to consume significantly more carbohydrates than those in the control group. Additionally, the researchers found that patients with a high CD activity index (CDAI > 150) had a higher amount of carbohydrates in their diet than patients in remission. In addition to carbohydrates, the effect of proteins on CD development is possible: for example, in the study [14], a positive correlation was shown between the increased consumption of animal protein and the occurrence of CD among Japanese residents.

### 2.2. Smoking

A study conducted in France showed that the proportion of the active disease course in CD patients who did not smoke was lower than in light smokers (up to 10 cigarettes per day) and heavy smokers (more than 10 cigarettes per day): 33% versus 38% and 41%, respectively [15]. In another study [16], smoking was also shown to increase the risk of surgery in CD patients: smokers had 20% higher risk than non-smokers. In addition to influencing the occurrence and progression of CD, smoking can affect the body’s sensitivity to the effects of drugs. The results of the analysis of clinical outcomes revealed that periodic use of infliximab was effective in 73% non-smoking patients and only in 22% among smokers [17]. The question was also investigated whether quitting smoking would have a beneficial effect on the disease course in CD patients. Among the group of patients who quit smoking (who were tested for nicotine content in the urine), the risk of exacerbation of the disease was lower than that of smokers, at the same time, there was no reliable difference between quit and non-smokers [18].

Several studies have shown that smoking increases the risk of CD development; however, the exact mechanisms of such influence remain unknown. Four possible smoking targets that may lead to CD were proposed: gastrointestinal microbiota, the gut immune system, intestinal epithelium integrity and epigenetic influence [19]. Approximately4500 compounds present in tobacco smoke, about 150 can have a toxic and carcinogenic effect on the organism, among which are dioxins that have a proven immunomodulatory effect [19]. Some studies provided indications for possible mechanisms of smoking effects on CD development. For instance, in a multifactorial study in which patients were stratified for disease severity, it was shown that the proportion of *Bacteroides*–*Prevotella* that are conditionally pathogenic bacteria was higher in smokers compared to non-smokers (38.8% versus 28.3%) [20]. In another study in patients with UC, smoking patients were shown to have higher intestinal permeability compared to non-smoking patients and a negative control group [21]. In a mouse model study, passive smoking was shown to increase the number of dendritic cells, increase chemokine expression and T-lymphocyte activation [22].

### 2.3. The Effect of Therapeutics on CD Development

An additional factor that increases the risk of CD development is the use of certain groups of therapeutic drugs: antibiotics, nonsteroidal anti-inflammatory drugs (NSAIDs) and contraceptives [23]. According to the currently used hypothesis, antibiotic intake in childhood causes a disruption of the body’s tolerance to gut microbiota, which can lead to the emergence of CD later in life [24]. Several studies were shown that antibiotic intake positively correlated with CD development [23]. There was also a positive association between women taking contraceptive pills and the risk of CD, with women who stopped consuming contraceptive pills having a reduced risk of CD [25]. The exact mechanisms of these drugs’ influence on the risk of CD occurrence are unknown. However, the effect intensifies under the action of estrogen, which enhances the immune response, and weakens under influence of progesterone, which acts as immunosuppressor [26]. A prospective cohort study of over 76,000 women nurses revealed an increased risk of CD in the study subjects who took NSAIDs for at least 15 days per month [27]. Moreover, another study in patients with IBD who were in remission, taking NSAIDs was associated with relapse within 9 days with a frequency of 15 to 30% [28].

### 2.4. Genetic Factors

The influence of genetic mutations on the development of CD has been fully proven: to date, more than 230 single nucleotide polymorphisms (SNPs) associated with IBD have been identified [5]. It was found that first degree relatives (FDR) of patients with IBD also have an increased risk of developing IBD: the incidence rate of CD was 7.77, and the degree of concordance in monozygous twins was 30–35% [29]. The main identified genetic risk factor for CD is the *NOD2* gene, which is expressed in the intestinal epithelial cells, intestinal mucosal lymphocytes, as well as in monocytes and macrophages [5]. This gene encodes a receptor that recognizes muramyl dipeptide (MDP), a component of peptidoglycan from the bacterial wall. NOD2 interaction with MDP triggers a signaling cascade that activates transcription of proinflammatory cytokines and, accordingly, activates innate immune response. The most frequent mutations in the *NOD2* gene responsible for CD development affect amino acids that are in the leucine-rich repeat-LRR domain, which is responsible for binding to MDP [5]. A hypothesis has been formulated, according to which, mutations in the *NOD2* gene lead to increased reproduction of intestinal bacteria, which causes enhanced immune response of the host [30]. Thus, the loss of regulation of gut microbiota mass and composition with disturbance of the immune system can trigger a sharp immune response that can evolve to the state of chronic inflammation. However, despite the fact that *NOD2* is the main genetic locus associated with CD risk, mutations in this locus cannot be considered as a necessary and sufficient condition for CD development, since they can occur in a population of healthy people with a frequency of 0.5–2%, and, at the same time, in 60–70% of patients with CD do not carry *NOD2* mutations [31].

Mutations in genes: *ATG16L1*, *LRRK2*, and *IRGM* that are involved in autophagy are also associated with CD risk [32]. Autophagy is a multistage process, orchestrated by various proteins, whereby its function is to destroy superfluous and dysfunctional organelles. A particular type of autophagy is mitophagy, which is specialized on the destruction of dysfunctional mitochondria. When mitophagy is disturbed, a large number of mitochondria and mitochondria-derived molecules accumulate in the cytosol. These molecules, that are normally sequestrated within the outer mitochondrial membrane, are recognized as endogenous antigens-DAMP (damage-associated molecular pattern). Like external antigens PAMP (pathogen-associated molecular patterns), DAMPs are able to trigger inflammatory immune response [33]. Disruption of the intestinal mucosa integrity can lead to impairment of gut microbiome regulation and inflammation triggering; therefore, mutations in genes controlling the permeability of the intestinal wall, such as *MUC19*, *ITLN1*, *FUT2*, and *XBP1*, were identified as additional risk factors for CD development [5]. A study using GWAS analysis described several variants of gene alleles encoding DNA-methyltransferases and other proteins taking part in epigenetic modifications associated with a risk of CD development [34]. In a study of zebrafish, inactivation or expression of the *uhrf1* gene responsible for DNA methylation resulted in IBD syndrome development [35].

### 2.5. Dysbiosis

The gut microbiota is the largest reservoir of bacteria in the human body, with the highest number of bacteria concentrated in the large intestine lumen. The bacterial count there can reach 1011–1012 cells/g of lumen contents [36]. The human genome includes approximately 23,000 genes, while the gut microbiome contains more than 3 million genes that have an important impact on human health [37]. The gut microbiota performs important functions for the body: digesting substrates that gastrointestinal enzymes cannot cope with, training the immune system tolerance, restraining pathogenic microorganisms’ growth, producing biologically active substances, such as butyrate, that supports the energy needs of enterocytes, facilitating the absorption of calcium and iron in the colon [38,39].

It has been proven that gut dysbiosis—a change in the qualitative and quantitative composition of the microbiome—is a common feature in IBD. However, it remains unclear whether this condition is associated with a cause or consequence of the inflammatory reaction development. According to the results of studies of gut microbiota among patients with IBD, changes were found associated with increased bacterial load and decreased bacterial species diversity [40]. A change in microbiota composition was also found in feces and intestinal mucosa samples from IBD patients. Moreover, the number of bacteria was significantly higher in the regions of the intestine with the greatest inflammation, in the colon and ileum, which proved the connection of dysbiosis with the development of inflammation in IBD [40].

A general pattern for the state of dysbiosis in IBD is a decrease in the number of bacterial species that are beneficial for the organism. Several studies reported a decrease in species diversity and the number of *Bacteroidetes* and *Firmicutes* types in CD patients that are dominant types for normal microbiota, for example, a decrease in the number of *Faecalibacteriumprausnitzii*, *Roseburia* or *Eubacterium* bacteria [38,41]. A decrease in *Bacteroides fragilis* was also found to be involved in the activation of T-regulatory cells having an anti-inflammatory effect [38]. Additionally, the number of *Bifidobacterium* type bacteria having important protective and metabolic functions for the organism was reduced [38]. At the same time, the number of conditionally pathogenic types of *Proteobacteria* (*Escherichia coli*, *Pasteurellaceae*), *Firmicutes* (*Veillonellaceae* and *Ruminococcusgnavus*) and *Fusobacterium* species is increasing [38]. In addition, it was shown that *Escherichia coli* bacteria were able to adhere on the intestinal wall and penetrated through the intestinal epithelial layer, also being found embedded and multiplied in macrophages, which caused an enhanced release of the inflammatory mediator TNFα [42].

## 3. Models of CD Pathogenesis Based on Mitochondrial Dysfunction

The effect of mitochondrial dysfunction in CD development can be divided into two distinct pathways. The first one is the impact of energy shortage through impaired ATP synthesis, which leads to disruption of a number of processes important for the proper functioning of the intestine. Among these processes are the differentiation of enterocytes, tight junction maintenance and butyrate oxidation. In this model, inflammation is formed on bacterial antigens. The second pathway is based not on an energy deficit in the intestinal cells, but on increased generation of reactive oxygen species (ROS) and disruption of mitophagy, which leads to the accumulation of defective mitochondria. This process is accompanied by the accumulation of components acting as internal antigens (DAMPs) and the activation of the inflammatory response.

### 3.1. Models of CD Pathogenesis Based on an Energy Deficit in the Intestine Cells

Energy production through ATP synthesis depends on a number of processes: the conversion of metabolite energy into the energy of chemical bonds of reduced NADH molecules, the transfer of electrons from NADH to the electron transport chain and ultimately to molecular oxygen, and pumping protons from the mitochondrial matrix through the inner mitochondrial membrane to the intermembrane space, which generates transmembrane proton gradient. The generated energy is used for phosphorylation of ADP molecules to form ATP [43].

At the molecular level, mitochondrial dysfunction manifests itself in disruption of the processes involved in mitochondrial energy production: loss of electrochemical potential on the inner mitochondrial membrane, disruption of electron transport chain transporters and a reduction in the key metabolites transported into the mitochondria [43]. These changes lead to a decrease in oxidative phosphorylation efficiency and ATP production, which, in turn, leads to energy deficiency in the affected cell. Mitochondrial dysfunction is a well-known feature of various chronic diseases associated with low-level sustained inflammation [44]. This hypothesis is confirmed by the presence of mitochondrial dysfunction in CD. Oxidative phosphorylation deficiency in complexes III and IV of the respiratory chain has been reported in such patients [45]. It can be speculated that, since the proper energy balance is crucial for the correct functioning of the intestinal cells and the controlling of intestinal wall permeability, mitochondrial dysfunction in these cells can contribute to CD development.

### 3.2. Mechanism of CD Pathogenesis Based on Impaired Enterocyte Differentiation

Intestinal epithelium is updated every 4–5 days, which requires the consumption of a significant amount of energy [46]. Intestinal epithelium consists of one layer of different cell types, among which are goblet cells, absorptive enterocytes, Paneth cells, and enteroendocrine cells [46]. All these cells originate as a result of differentiation of intestinal stem cells. Lack of ATP leads to impaired differentiation of intestinal epithelial cells (IEC) [46]. Of particular importance is the disruption of the Paneth cells formation, which play the role of primary protection of the intestine, releasing antimicrobial molecules, such as defensins [47]. A decrease in the number of Paneth cells can lead to intestinal dysbiosis, which is a known characteristic of CD. If dysbiosis reaches a certain level, it may lead to the initiation of a strong inflammatory reaction that develops into chronic inflammation.

The vulnerability of Paneth cells was demonstrated in several studies. One of such studies reported that mice defective for the *phb1* gene encoding the main component of the internal mitochondrial membrane developed spontaneous inflammation in the ileum, preceded by mitochondrial dysfunction observed in Paneth cells [9]. It was also shown that mitochondrial dysfunction could lead to differentiation of dysfunctional Paneth cells from the intestinal stem cells, resulting in relapses in patients with CD [48]. Additionally, expression of some IEC differentiation genes was found to be impaired upon the development of inflammatory response in the intestine [46]. In a mouse model, increased ATP production as a result of increased oxidative phosphorylation activity was shown to protect mice from colitis, which was induced by administration of sodium dextran sulfate and trinitrobenzene sulfonate, and also contributed to increased enterocyte proliferation, which proved a positive effect of ATP generation on the rate of intestinal epithelium renewal [49].

### 3.3. Mechanism of CD Pathogenesis Based on the Disruption of Tight Junction Integrity

The intestinal epithelium acts as a protective barrier, preventing penetration of bacteria, debris and other substances from the intestinal lumen into the intestinal wall [50]. The key role in maintaining the integrity of the intestinal barrier is played by tight junctions, which are formed by proteins, mainly claudins and occludins, connecting adjacent intestinal epithelial cells and delimiting intestinal lumen from lamina propria [51]. Since maintaining the integrity of tight junctions requires ATP energy [46], in case of its deficiency, intestinal permeability increases, which leads to the penetration of bacterial antigens through the epithelial layer from the intestinal lumen into lamina propria, where intestinal immune cells are concentrated. The inflow of antigens also causes increased proliferation and inflow of immune cells, leading to the inflammatory response activation. It was shown that mitochondrial damage could influence increased intestinal barrier permeability for pathogens [52].

### 3.4. Mechanism of CD Pathogenesis Based on Impaired Butyrate Oxidation and Its Deficiency

One current hypothesis is based on the observation that IBD is characterized by a state of energy deficiency with a change of metabolism in the intestinal epithelial cells [53]. Butyrate is the main energy source of colon epithelial cells, providing more than 70% of energy demand [46]. Butyrate undergoes cleavage in the mitochondrial matrix of colonocytes in the process of β-oxidation of fatty acids [46]. Butyrate is a nutrient that is contained in food and is also formed by intestinal bacteria as a by-product of fermentation of dietary fibers [54]. Since butyrate is the main source of energy for colonocytes, the disruption of its oxidation in the mitochondria leads to a lack of energy to perform important processes in the intestine functioning described above, which, in turn, leads to the development of severe gut dysbiosis and activation of the inflammatory reaction. In the study in a model of experimental animals, it was shown that inflammation of the intestinal mucosa occurred when inhibiting butyrate oxidation [55]. It was also shown that pharmacological inhibition of β-oxidation of fatty acids in the intestine led to the development of colitis in mice [56].

**Figure 1:**
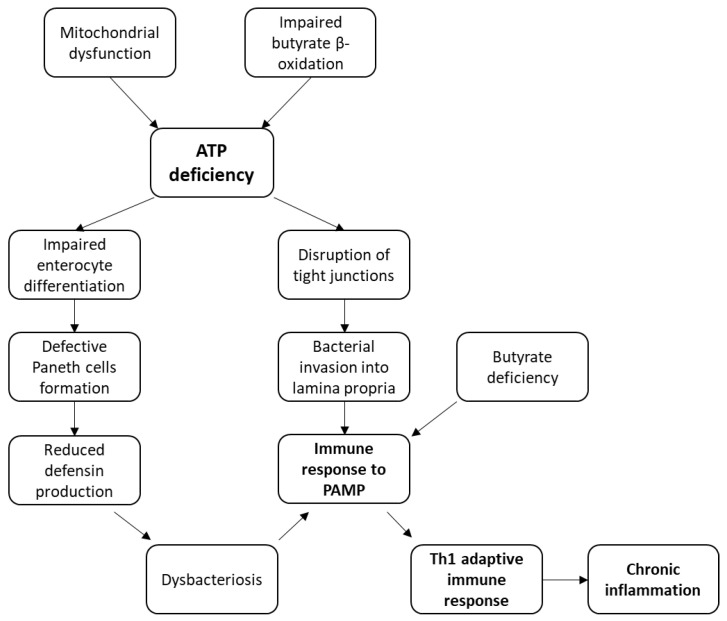
Models of CD pathogenesis with inflammation induction by PAMP.

In addition to being an energy source, butyrate is an anti-inflammatory agent that induces a mediated antimicrobial action. Thus, butyrate inhibits the action of NF-kB, IFNγ, pro-inflammatory cytokines IL2, IL6 and IL8, reduces the recruitment of macrophages and neutrophils, enhances the action of an antimicrobial protein catelicidin, and increases the expression of mucin, which also has an antimicrobial effect [57,58,59,60]. Accordingly, if dietary butyrate is lacking, or if butyrate deficiency is caused by a decrease inthe gut microbiome species producing it, no inflammatory response deterrent remains except for ATP deficiency. This option is not directly related to the mitochondria, but can be considered in the framework of the mitochondrial model of CD development. Scheme of these models are depicted on Figure 1.

### 3.5. Immune Response to Bacterial Antigens in CD

According to the pathogenetic models described above, the key outcome leading to CD development is the initiation of an immune response to bacterial antigens, which leads to the development of chronic inflammation. Gut-associated lymphoid tissue (GALT) plays a controversial role in the pathology development: in the case of infection, the proximity of immune cells is a positive factor; however, in the case of IBD, it has negative influence. The primary response to intestinal dysbiosis is initiation of innate immune response. Innate immunity leukocytes represented by neutrophils, macrophages, and dendritic cells recognize bacteria through interaction with PAMPs, which are present in most microorganisms [61]. Examples of conservative PAMPs are lipopolysaccharides (LPS), peptidoglycan, flagellin and bacterial nucleic acids [61]. The key receptors recognizing bacterial PAMPs are TLR and NRL receptors that are expressed not only on immune, but also on intestinal epithelial cells. Therefore, enterocytes can also directly activate the inflammatory response [61]. In addition, M-cells can be engaged in the capture of bacterial antigens in the intestine. They transport antigens to Peyer’s patches-GALT structural elements, where they are selected by dendritic cells or destroyed by macrophages [62].

Interaction of PAMPs with the innate immunity receptor leads to activation of inflammatory cascades involving pro-inflammatory transcription factor NF-κB and IL-1β, as well as other inflammatory cytokines highly present in the intestine in CD: IL-12, IL-17, IL-18, TNF-α and IFN-γ. Activated antigen presenting cells (APCs) produce IL-12 and IL-18 and induce polarized differentiation of CD4+ T lymphocytes along the Th1 pathway [63]. That, in turn, further increases the release of proinflammatory cytokines that stimulate APCs to produce new types of proinflammatory cytokines, such as IL-1, IL-6, and IL-8. Therefore, a self-perpetuating inflammatory response is formed that leads to chronic inflammation development.

### 3.6. Models of CD Pathogenesis Based on ROS Production and Mitophagy Disorders

The by-product of electron transport chain (ETC) functioning in the mitochondria are reactive oxygen species (ROS): molecules with increased reactivity due to the presence of unpaired electron at the external electronic level [64]. Under normal physiological conditions, the amount of ROS generated from the total amount of oxygen consumed by cells is around 2%; however, in a pathological state, ROS release increases [65]. The increase in ROS production is associated with disturbances in the respiratory complexes functioning, with increased proton leakage and excessive oxygen consumption, accompanied by a further increase in ROS generation [66]. ROS molecules readily interact with various biological molecules, including proteins, lipids and nucleic acids, which leads to damaging effects [65]. Inside the mitochondria, ROS damage phospholipids of the outer and inner mitochondrial membranes, especially cardiolipin, which is highly sensitive to ROS. Oxidative destruction of the lipids of the internal mitochondrial membrane leads to the reverse transfer of protons to the mitochondrial matrix; therefore, decreasing the electrochemical gradient [46]. As a result of mitochondrial damage, various mitochondrial molecules enter the cytoplasm. Some of them are highly immunogenic and can act as internal antigens DAMPs: succinate, cardiolipin, N-formyl peptides, mitochondrial DNA (mtDNA) and mitochondrial transcription factor A (TFAM), while others, such as cytochrome *c*, can act as signals to cell death [67]. Damaged mitochondria are normally isolated by mitophagy into membranous structures that are then fused with lysosomes for degradation by lysosomal enzymes. Mitophagy is the cellular process that selectively removes old, damaged and dysfunctional mitochondria by sequestration [68]. Mitophagy, along with other mitochondrial dynamics processes such as mitochondrial fusion, fission, and transport, plays an important role in maintaining normal cell homeostasis. Many proteins, molecular intermediates, activators and inhibitors participate in coordination of the mitophagy process, so the regulation of mitophagy is a complex process and can be disrupted, leading to the formation of a pool of defective and dysfunctional mitochondria.

**Figure 2:**
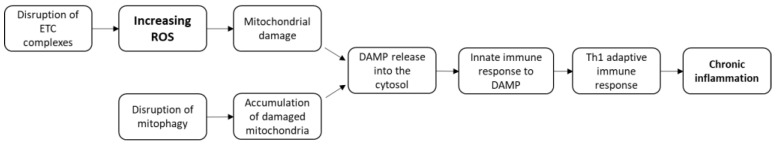
Models of CD pathogenesis with inflammation induction by DAMP.

Increasing the number of defective mitochondria leads to even greater DAMP generation, which triggers an inflammatory response. Thus, mtDNA, entering the cytosol, activates the inflammasome NLRP3, which, in turn, leads to the activation of caspase-1 and the production of proinflammatory cytokines IL-1β and IL-18 [33,67]. Additionally, cytosolic mtDNA can also directly activate AIM2-bound inflammasome [67]. Thus, mtDNA acts as an intracellular activator of the inflammatory reaction. In addition, inflammation activation is also possible when the immune system cells are directly affected by the extracellular molecules DAMP. So, extracellular ATP, can activate inflammasome NLRP3 and promote, thus, secretion of IL-1β and IL-18 in macrophages [69]. An additional role of ATP is to attract neutrophils to the inflammation site [69]. Another mitochondrial DAMP, succinate, which is an intermediate in the tricarboxylic acid cycle, can also be secreted into the extracellular space when mitochondria are damaged. Extracellular succinate enhances antigen-dependent activation of T helper cells by interaction with dendritic cell receptor GPR91 [70]. The subsequent development of the inflammatory response is similar to the scheme described previously, including the polarization of CD4+ T lymphocytes towards Th1 phenotype and the formation of an irreversible reaction involving a large number of inflammatory molecules and immune cells, which leads to the establishment chronic inflammation state. These models are schematically depicted in Figure 2.

## 4. Future Directions

Mitochondria are currently considered not only as energy “plants” of the cell, but also as important players in the processes of immunomodulation and regulation of cellular homeostasis. The idea of the mitochondrial dysfunction influencing the development of chronic inflammation is not new and is used as a hypothesis for explaining the pathogenesis of various chronic diseases, fatigue and aging. However, the pathogenesis models discussed in this review are specifically focused on the development of chronic inflammation in CD. Some aspects of these models are supported by studies, but more detailed investigation is required to fully prove their feasibility, with full reproduction of these pathogenesis mechanisms in laboratory animals. Moreover, an important step will be the finding of specific mitochondrial targets from which mitochondrial dysfunction and the development of subsequent inflammation begin.

As described in the previous chapters, mitochondrial dysfunction was shown to play a prominent role in CD pathogenesis, especially in Paneth cells, where mitochondrial function is crucial to maintain cellular functionality. Correspondingly, mitochondria-targeting therapies are being evaluated to reduce the severity of CD manifestations in model organisms. One of such approaches is the use of mitochondrial ROS scavengers that allow reducing the mitochondrial damage-associated oxidative stress. One recent study tested a mitochondrial antioxidant, Mito-Tempo, on ex vivo biopsy material from CD patients. The study confirmed the presence of mitochondrial damage in ileal mucosal biopsy samples form CD patients as compared with non-IBD patients that manifested itself at the phenotype and transcriptome levels. Treatment with Mito-Tempo allowed restoring the expression of altered genes to non-IBD levels, including genes involved in inflammation (IL-17/IL-23), lipid metabolism and apoptosis regulation [71]. These results are promising for potential future use of mitochondrial antioxidants for CD treatment, but more studies are needed to validate these results in animal models. One such model is the TNF^ΔARE^ mouse model bearing a deletion of adenylate/uridylate-rich elements (AREs) in TNF mRNA. Such animals present with inflammation-associated mitochondrial dysfunction in Paneth cells and develop spontaneous ileitis. Promoting mitochondrial respiration through dichloroacetate-mediated glycolysis inhibition was shown to improve the function of intestinal stem cells from these animals. Therefore, suppressing glycolysis to restore mitochondrial metabolic balance may be explored as one of the potential therapeutic approaches to CD [48,72].

Another recent study tested the effect of Olaparib, the inhibitor of poly(ADP-ribose) polymerase-1 (PARP-1) currently used for cancer therapy on the manifestations of chemically-induced experimental colitis in mice. PARP-1 plays an important role in inflammation development through mitochondrial dysfunction. Its inhibition reduced the inflammation markers and restore the intestinal barrier function in affected animals. In colon epithelial cell monolayer treated with hydrogen peroxide, Olaparib helped preserving barrier integrity and alleviated morphological changes. It also improved cellular mitochondrial function under oxidative stress conditions. Together, these results indicate the possible use of PARP-1 inhibitor to treat CD and warrant further preclinical and clinical studies [73].

Another interesting model for developing novel CD therapies is the recently described patient-specific human intestinal organoid (HIO) [74]. This model, consistent with patient-derived ileal cells, is characterized by the expression of mitochondrial and extracellular matrix genes reflecting a human situation. It makes possible studying the expression pattern and morphology and their alterations in response to different potential therapeutic agents through RNA sequencing and immunostaining techniques. Importantly, HIO is a patient-specific modelling approach, which can potentially be used for the development of personalized treatments. In a recent study, the effects of butyrate and eicosatetraynoic acid were tested in such model showing promising results [74].

As we hoped to demonstrate in this review, CD pathogenesis is complex, with several pathogenic pathways that can complement and overlap each other. Together with other inflammatory bowel diseases, CD is currently regarded as a pathologic condition associated with chronic inflammation, in which mitochondrial dysfunction plays a prominent role. Correspondingly, mitochondria-targeting therapies are being developed and evaluated for these diseases [75]. One of the great challenges of this research is the creation of reliable preclinical models that would allow identifying reliable molecular targets for therapeutic effects. Such models should reflect, at least to a certain degree, the complexity of pathological processes taking place in the intestinal cells and tissues. The strategy of developing anti-CD drugs aimed at the initial stages of disease pathogenesis rather than its final symptomatic stages involving the inflammatory reaction in the gastrointestinal tract, are especially interesting. Such an approach would help to reduceside effects caused by anti-inflammatory drugs and increase the therapy effectiveness. In this review, we strived to show that despite formal understanding of the etiological factors underlying CD development, many unexplored stages of CD pathogenesis remain, the disclosure of which will help in the fight against this disease.

## 5. Conclusions

We propose four pathogenetic models for the development of CD, with mitochondrial dysfunction development as a central event, and one model based on the lack of butyrate, an important anti-inflammatory mediator, in the intestine. Models associated with mitochondrial dysfunction can be divided into two groups: the first group is based on disorders in the vital functions of enterocytes caused by energy deficiency, where bacterial PAMPs act as antigens that cause an inflammatory reaction; the second group is associated with increased production of ROS and impaired mitophagy, leading to the release of mitochondrial DAMPs playing the role of antigens that initiate inflammation.
